# *catena*-Poly[[[2,6-bis­(pyrazol-1-yl-κ*N* ^2^)pyridine-κ*N* ^1^](nitrato-κ^2^ *O*,*O*′)cadmium(II)]-μ-thio­cyanato-κ^2^ *N*:*S*]

**DOI:** 10.1107/S1600536808032297

**Published:** 2008-10-11

**Authors:** Zhong Nian Yang, Ting Ting Sun

**Affiliations:** a Department of Chemistry and Chemical Engineering, Binzhou University, Binzhou 256603, People’s Republic of China; b Department of Chemistry, Shandong Normal University, Jinan 250014, People’s Republic of China

## Abstract

In the title crystal structure, [Cd(NCS)(NO_3_)(C_11_H_9_N_5_)]_*n*_, the unique Cd^II^ ion is coordinated in a distorted penta­gonal–bipyramidal environment. The axial thio­cyanate ligands act in a μ_1,3_-bridging mode to connect symmetry-related Cd^II^ ions into one-dimensional chains along [010]. In addition, there are inter­molecular C—H⋯O contacts between chains.

## Related literature

For background information, see: Halcrow (2005 ▶); Shi *et al.* (2006 ▶). 
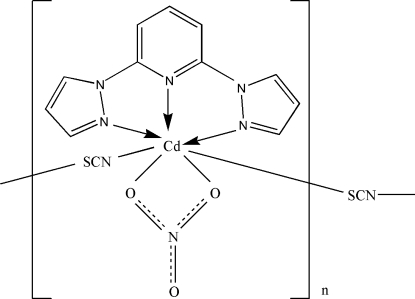


## Experimental

### 

#### Crystal data

[Cd(NCS)(NO_3_)(C_11_H_9_N_5_)]
*M* *_r_* = 443.72
Monoclinic, 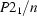

*a* = 8.4161 (15) Å
*b* = 11.817 (2) Å
*c* = 15.631 (3) Å
β = 99.673 (2)°
*V* = 1532.5 (5) Å^3^
*Z* = 4
Mo *K*α radiation
μ = 1.59 mm^−1^
*T* = 298 (2) K
0.18 × 0.15 × 0.11 mm

#### Data collection

Bruker SMART APEX CCD diffractometer
Absorption correction: multi-scan (*SADABS*; Sheldrick, 1996 ▶) *T*_min_ = 0.763, *T*_max_ = 0.845
8813 measured reflections
3335 independent reflections
2710 reflections with *I* > 2σ(*I*)
*R*_int_ = 0.034

#### Refinement

*R*[*F* ^2^ > 2σ(*F* ^2^)] = 0.032
*wR*(*F* ^2^) = 0.074
*S* = 1.02
3335 reflections
217 parameters
1 restraint
H-atom parameters constrained
Δρ_max_ = 0.53 e Å^−3^
Δρ_min_ = −0.35 e Å^−3^

### 

Data collection: *SMART* (Bruker, 2007 ▶); cell refinement: *SAINT* (Bruker, 2007 ▶); data reduction: *SAINT*; program(s) used to solve structure: *SHELXTL* (Sheldrick, 2008 ▶); program(s) used to refine structure: *SHELXTL*; molecular graphics: *SHELXTL*; software used to prepare material for publication: *SHELXTL*.

## Supplementary Material

Crystal structure: contains datablocks I, global. DOI: 10.1107/S1600536808032297/lh2703sup1.cif

Structure factors: contains datablocks I. DOI: 10.1107/S1600536808032297/lh2703Isup2.hkl

Additional supplementary materials:  crystallographic information; 3D view; checkCIF report

## Figures and Tables

**Table:** 

Cd1—N6	2.279 (3)
Cd1—N1	2.346 (3)
Cd1—O3	2.361 (2)
Cd1—N5	2.379 (3)
Cd1—N3	2.388 (2)
Cd1—O2	2.495 (2)
Cd1—S1^i^	2.7447 (9)

**Table:** 

N6—Cd1—N1	93.43 (12)
N6—Cd1—O3	90.12 (11)
N1—Cd1—O3	136.31 (9)
N6—Cd1—N5	89.13 (10)
N1—Cd1—N5	134.53 (10)
O3—Cd1—N5	89.01 (9)
N6—Cd1—N3	100.47 (10)
N1—Cd1—N3	67.50 (9)
O3—Cd1—N3	153.74 (9)
N5—Cd1—N3	67.41 (9)
N6—Cd1—O2	81.17 (9)
N1—Cd1—O2	85.22 (9)
O3—Cd1—O2	52.36 (8)
N5—Cd1—O2	139.77 (9)
N3—Cd1—O2	152.71 (9)
N6—Cd1—S1^i^	173.33 (8)
N1—Cd1—S1^i^	86.04 (7)
O3—Cd1—S1^i^	85.71 (6)
N5—Cd1—S1^i^	95.98 (6)
N3—Cd1—S1^i^	85.49 (6)
O2—Cd1—S1^i^	92.16 (6)

Symmetry code: (i) 
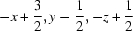
.

**Table 2:** Hydrogen-bond geometry (Å, °)

*D*—H⋯*A*	*D*—H	H⋯*A*	*D*⋯*A*	*D*—H⋯*A*
C3—H3⋯O1^ii^	0.93	2.50	3.412 (5)	167
C4—H4⋯O2^iii^	0.93	2.47	3.370 (4)	164
C7—H7⋯O3^iv^	0.93	2.52	3.312 (5)	143
C10—H10⋯S1^iv^	0.93	2.83	3.723 (4)	160

Symmetry codes: (ii) 
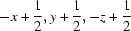
; (iii) 
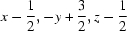
; (iv) 
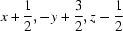
.

**Fig. 1.**
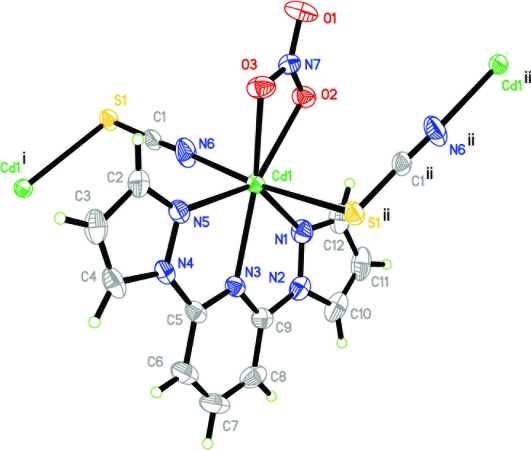
View of part of the structure of (I), with displacement ellipsoids drawn at the 30% probability level. [Symmetry codes: (i) -x + 3/2, y + 1/2, -z + 1/2; (ii) -x + 3/2, y - 1/2, -z + 1/2.]

**Fig. 2.**
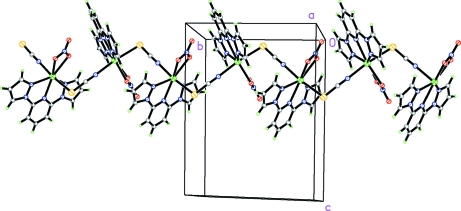
Part of the one-dimensional chain of (I).

## supplementary crystallographic information

### Comment

Both the 2,6-bis(pyrazolyl)pyridine and thiocyanate ligands play an important role in modern coordination chemistry (Halcrow 2005; Shi *et al.* 2006), and our interest in complexes formed with these ligands led us to prepare the title complex and determine its crystal structure (I).

As shown in Fig. 1 the Cd^II^ ion is coordinated in a distorted pentagonal–bipyramidal environment with the 2,6-bis(pyrazolyl)pyridine and nitrate anion acting as chelating tridentate and bidentate ligands, respectively. The axial thiocyantate ligands bridge symmetry-related Cd^II^ ions [with a Cd···Cd separation of 6.1817 (10) Å] to form a one-dimensional `zigzag' chain along the *b* axis (Fig. 2). In addition, the crystal structure contains C—H···O and C—H···S short contacts between chains.

### Experimental

A 15 ml methanol solution containing 2,6-bis(pyrazolyl)pyridine (0.4140 g, 0.196 mmol) was added to 8 ml H_2_O solution of Cd(NO_3_)_2_6H_2_O (0.0689 g, 0.200 mmol) and NaSCN (0.0324 g, 0.400 mmol), and the mixture was stirred for a few minutes. Colorless single crystals were obtained after the filtrate was allowed to stand at room temperature for a month.

### Refinement

All H atoms were placed in calculated positions with C—H = 0.93 Å and refined as riding with *U*_iso_(H) = 1.2*U*_eq_(C).

### Crystal data

**Table:**

[Cd(NCS)(NO_3_)(C_11_H_9_N_5_)]	*F*(000) = 872
*M**_r_* = 443.72	*D*_x_ = 1.923 Mg m^−^^3^
Monoclinic, *P*2_1_/*n*	Mo *K*α radiation, λ = 0.71073 Å
Hall symbol: -P 2yn	Cell parameters from 2732 reflections
*a* = 8.4161 (15) Å	θ = 2.2–24.8°
*b* = 11.817 (2) Å	µ = 1.59 mm^−^^1^
*c* = 15.631 (3) Å	*T* = 298 K
β = 99.673 (2)°	Block, colourless
*V* = 1532.5 (5) Å^3^	0.18 × 0.15 × 0.11 mm
*Z* = 4	

### Data collection

**Table:**

Bruker SMART APEX CCD diffractometer	3335 independent reflections
Radiation source: fine-focus sealed tube	2710 reflections with *I* > 2σ(*I*)
graphite	*R*_int_ = 0.034
φ and ω scans	θ_max_ = 27.0°, θ_min_ = 2.2°
Absorption correction: multi-scan (SADABS; Sheldrick, 1996)	*h* = −10→7
*T*_min_ = 0.763, *T*_max_ = 0.845	*k* = −15→14
8813 measured reflections	*l* = −19→19

### Refinement

**Table:**

Refinement on *F*^2^	Primary atom site location: structure-invariant direct methods
Least-squares matrix: full	Secondary atom site location: difference Fourier map
*R*[*F*^2^ > 2σ(*F*^2^)] = 0.032	Hydrogen site location: inferred from neighbouring sites
*wR*(*F*^2^) = 0.074	H-atom parameters constrained
*S* = 1.02	*w* = 1/[σ^2^(*F*_o_^2^) + (0.0324*P*)^2^] where *P* = (*F*_o_^2^ + 2*F*_c_^2^)/3
3335 reflections	(Δ/σ)_max_ = 0.001
217 parameters	Δρ_max_ = 0.53 e Å^−^^3^
1 restraint	Δρ_min_ = −0.35 e Å^−^^3^

### Special details

**Table:**

Geometry. All e.s.d.'s (except the e.s.d. in the dihedral angle between two l.s. planes) are estimated using the full covariance matrix. The cell e.s.d.'s are taken into account individually in the estimation of e.s.d.'s in distances, angles and torsion angles; correlations between e.s.d.'s in cell parameters are only used when they are defined by crystal symmetry. An approximate (isotropic) treatment of cell e.s.d.'s is used for estimating e.s.d.'s involving l.s. planes.
Refinement. Refinement of *F*^2^ against ALL reflections. The weighted *R*-factor *wR* and goodness of fit *S* are based on *F*^2^, conventional *R*-factors *R* are based on *F*, with *F* set to zero for negative *F*^2^. The threshold expression of *F*^2^ > σ(*F*^2^) is used only for calculating *R*-factors(gt) *etc*. and is not relevant to the choice of reflections for refinement. *R*-factors based on *F*^2^ are statistically about twice as large as those based on *F*, and *R*- factors based on ALL data will be even larger.

### Fractional atomic coordinates and isotropic or equivalent isotropic displacement parameters (Å^2^)

**Table:**

	*x*	*y*	*z*	*U*_iso_*/*U*_eq_	
C1	0.8278 (4)	0.8449 (3)	0.3362 (2)	0.0413 (8)	
C2	0.3464 (4)	0.7893 (3)	0.1478 (2)	0.0507 (9)	
H2	0.3042	0.7689	0.1968	0.061*	
C3	0.2736 (5)	0.8650 (3)	0.0850 (3)	0.0582 (11)	
H3	0.1769	0.9034	0.0840	0.070*	
C4	0.3722 (5)	0.8708 (3)	0.0265 (3)	0.0550 (10)	
H4	0.3569	0.9151	−0.0234	0.066*	
C5	0.6374 (4)	0.7813 (2)	0.01605 (19)	0.0399 (8)	
C6	0.6570 (5)	0.8277 (3)	−0.0627 (2)	0.0558 (10)	
H6	0.5765	0.8708	−0.0955	0.067*	
C7	0.8005 (6)	0.8073 (3)	−0.0903 (2)	0.0654 (12)	
H7	0.8187	0.8386	−0.1423	0.078*	
C8	0.9175 (5)	0.7420 (3)	−0.0429 (2)	0.0596 (11)	
H8	1.0153	0.7286	−0.0612	0.071*	
C9	0.8833 (4)	0.6968 (3)	0.0337 (2)	0.0428 (8)	
C10	1.1325 (5)	0.5770 (3)	0.0743 (3)	0.0666 (12)	
H10	1.1804	0.5861	0.0253	0.080*	
C11	1.1898 (5)	0.5134 (3)	0.1447 (3)	0.0705 (12)	
H11	1.2834	0.4701	0.1538	0.085*	
C12	1.0793 (5)	0.5266 (3)	0.2001 (3)	0.0633 (11)	
H12	1.0885	0.4923	0.2543	0.076*	
Cd1	0.69811 (3)	0.631846 (17)	0.194115 (13)	0.03553 (9)	
N1	0.9587 (4)	0.5939 (2)	0.16692 (19)	0.0501 (7)	
N2	0.9920 (4)	0.6253 (2)	0.08807 (19)	0.0468 (7)	
N3	0.7484 (3)	0.7163 (2)	0.06248 (15)	0.0370 (6)	
N4	0.4991 (3)	0.8004 (2)	0.05286 (16)	0.0389 (6)	
N5	0.4831 (3)	0.7503 (2)	0.12873 (16)	0.0416 (6)	
N6	0.7831 (5)	0.7729 (3)	0.29035 (19)	0.0716 (12)	
N7	0.6367 (3)	0.5128 (2)	0.34211 (16)	0.0410 (6)	
O1	0.6028 (3)	0.4666 (2)	0.40633 (16)	0.0704 (8)	
O2	0.7776 (3)	0.5144 (2)	0.32709 (14)	0.0483 (6)	
O3	0.5296 (3)	0.5612 (2)	0.28825 (14)	0.0552 (6)	
S1	0.89559 (11)	0.94486 (6)	0.40588 (5)	0.0430 (2)	

### Atomic displacement parameters (Å^2^)

**Table:**

	*U*^11^	*U*^22^	*U*^33^	*U*^12^	*U*^13^	*U*^23^
C1	0.049 (2)	0.0320 (17)	0.0409 (17)	0.0047 (15)	0.0013 (15)	0.0078 (14)
C2	0.049 (2)	0.0420 (19)	0.061 (2)	0.0077 (17)	0.0092 (18)	−0.0019 (16)
C3	0.042 (2)	0.045 (2)	0.083 (3)	0.0052 (17)	−0.007 (2)	−0.0069 (19)
C4	0.056 (2)	0.0379 (19)	0.063 (2)	0.0029 (18)	−0.015 (2)	0.0093 (16)
C5	0.052 (2)	0.0279 (15)	0.0362 (16)	−0.0140 (15)	−0.0035 (15)	0.0005 (13)
C6	0.074 (3)	0.048 (2)	0.0410 (19)	−0.017 (2)	−0.0016 (19)	0.0085 (16)
C7	0.094 (3)	0.064 (3)	0.0368 (19)	−0.031 (3)	0.007 (2)	0.0046 (18)
C8	0.069 (3)	0.061 (2)	0.055 (2)	−0.026 (2)	0.029 (2)	−0.0149 (19)
C9	0.050 (2)	0.0376 (18)	0.0404 (17)	−0.0173 (17)	0.0064 (16)	−0.0060 (14)
C10	0.047 (2)	0.063 (3)	0.095 (3)	−0.014 (2)	0.027 (2)	−0.032 (2)
C11	0.041 (2)	0.053 (2)	0.115 (4)	0.005 (2)	0.006 (2)	−0.024 (3)
C12	0.047 (2)	0.057 (2)	0.080 (3)	0.010 (2)	−0.007 (2)	−0.010 (2)
Cd1	0.04344 (16)	0.03132 (14)	0.03132 (13)	0.00301 (10)	0.00483 (10)	0.00214 (9)
N1	0.0439 (18)	0.0509 (16)	0.0541 (18)	0.0074 (15)	0.0047 (14)	0.0011 (14)
N2	0.0382 (17)	0.0452 (16)	0.0589 (18)	−0.0105 (13)	0.0133 (14)	−0.0136 (13)
N3	0.0421 (17)	0.0308 (13)	0.0372 (13)	−0.0067 (12)	0.0037 (12)	0.0000 (11)
N4	0.0430 (17)	0.0294 (13)	0.0402 (14)	−0.0014 (12)	−0.0045 (12)	0.0026 (11)
N5	0.0474 (18)	0.0340 (14)	0.0419 (15)	−0.0001 (13)	0.0032 (13)	0.0025 (11)
N6	0.116 (3)	0.0365 (17)	0.0525 (18)	0.0005 (18)	−0.015 (2)	−0.0097 (14)
N7	0.0462 (18)	0.0433 (15)	0.0339 (14)	0.0003 (14)	0.0076 (13)	−0.0010 (12)
O1	0.078 (2)	0.0841 (19)	0.0511 (15)	−0.0113 (16)	0.0165 (14)	0.0281 (14)
O2	0.0494 (15)	0.0532 (15)	0.0416 (11)	0.0051 (12)	0.0056 (11)	0.0075 (9)
O3	0.0491 (15)	0.0758 (17)	0.0407 (13)	0.0098 (13)	0.0080 (11)	0.0087 (12)
S1	0.0561 (6)	0.0331 (4)	0.0364 (4)	−0.0018 (4)	−0.0015 (4)	−0.0006 (3)

### Geometric parameters (Å, °)

**Table:**

C1—N6	1.135 (4)	C10—C11	1.352 (6)
C1—S1	1.642 (4)	C10—N2	1.362 (5)
C2—N5	1.319 (4)	C10—H10	0.9300
C2—C3	1.391 (5)	C11—C12	1.383 (6)
C2—H2	0.9300	C11—H11	0.9300
C3—C4	1.336 (6)	C12—N1	1.325 (4)
C3—H3	0.9300	C12—H12	0.9300
C4—N4	1.362 (4)	Cd1—N6	2.279 (3)
C4—H4	0.9300	Cd1—N1	2.346 (3)
C5—N3	1.327 (4)	Cd1—O3	2.361 (2)
C5—C6	1.383 (4)	Cd1—N5	2.379 (3)
C5—N4	1.400 (4)	Cd1—N3	2.388 (2)
C6—C7	1.370 (6)	Cd1—O2	2.495 (2)
C6—H6	0.9300	Cd1—S1^i^	2.7447 (9)
C7—C8	1.367 (5)	N1—N2	1.360 (4)
C7—H7	0.9300	N4—N5	1.352 (3)
C8—C9	1.385 (5)	N7—O1	1.218 (3)
C8—H8	0.9300	N7—O2	1.247 (3)
C9—N3	1.310 (4)	N7—O3	1.262 (3)
C9—N2	1.418 (4)	S1—Cd1^ii^	2.7447 (9)
			
N6—C1—S1	177.5 (3)	O3—Cd1—N5	89.01 (9)
N5—C2—C3	111.3 (4)	N6—Cd1—N3	100.47 (10)
N5—C2—H2	124.3	N1—Cd1—N3	67.50 (9)
C3—C2—H2	124.3	O3—Cd1—N3	153.74 (9)
C4—C3—C2	105.4 (4)	N5—Cd1—N3	67.41 (9)
C4—C3—H3	127.3	N6—Cd1—O2	81.17 (9)
C2—C3—H3	127.3	N1—Cd1—O2	85.22 (9)
C3—C4—N4	107.9 (3)	O3—Cd1—O2	52.36 (8)
C3—C4—H4	126.1	N5—Cd1—O2	139.77 (9)
N4—C4—H4	126.1	N3—Cd1—O2	152.71 (9)
N3—C5—C6	122.5 (4)	N6—Cd1—S1^i^	173.33 (8)
N3—C5—N4	115.2 (3)	N1—Cd1—S1^i^	86.04 (7)
C6—C5—N4	122.3 (3)	O3—Cd1—S1^i^	85.71 (6)
C7—C6—C5	117.0 (4)	N5—Cd1—S1^i^	95.98 (6)
C7—C6—H6	121.5	N3—Cd1—S1^i^	85.49 (6)
C5—C6—H6	121.5	O2—Cd1—S1^i^	92.16 (6)
C8—C7—C6	121.4 (4)	C12—N1—N2	105.0 (3)
C8—C7—H7	119.3	C12—N1—Cd1	136.2 (3)
C6—C7—H7	119.3	N2—N1—Cd1	116.9 (2)
C7—C8—C9	116.8 (4)	N1—N2—C10	110.1 (3)
C7—C8—H8	121.6	N1—N2—C9	119.7 (3)
C9—C8—H8	121.6	C10—N2—C9	130.1 (4)
N3—C9—C8	123.2 (3)	C9—N3—C5	119.0 (3)
N3—C9—N2	114.0 (3)	C9—N3—Cd1	120.8 (2)
C8—C9—N2	122.8 (3)	C5—N3—Cd1	120.2 (2)
C11—C10—N2	107.8 (4)	N5—N4—C4	110.1 (3)
C11—C10—H10	126.1	N5—N4—C5	120.2 (2)
N2—C10—H10	126.1	C4—N4—C5	129.6 (3)
C10—C11—C12	105.1 (4)	C2—N5—N4	105.3 (3)
C10—C11—H11	127.4	C2—N5—Cd1	137.6 (2)
C12—C11—H11	127.4	N4—N5—Cd1	117.0 (2)
N1—C12—C11	111.9 (4)	C1—N6—Cd1	177.7 (3)
N1—C12—H12	124.0	O1—N7—O2	121.5 (3)
C11—C12—H12	124.0	O1—N7—O3	120.9 (3)
N6—Cd1—N1	93.43 (12)	O2—N7—O3	117.6 (3)
N6—Cd1—O3	90.12 (11)	N7—O2—Cd1	91.99 (17)
N1—Cd1—O3	136.31 (9)	N7—O3—Cd1	98.02 (19)
N6—Cd1—N5	89.13 (10)	C1—S1—Cd1^ii^	99.61 (11)
N1—Cd1—N5	134.53 (10)		
			
N5—C2—C3—C4	−0.1 (4)	N6—Cd1—N3—C5	−87.3 (2)
C2—C3—C4—N4	0.5 (4)	N1—Cd1—N3—C5	−176.7 (2)
N3—C5—C6—C7	−2.4 (5)	O3—Cd1—N3—C5	24.9 (3)
N4—C5—C6—C7	177.1 (3)	N5—Cd1—N3—C5	−2.70 (19)
C5—C6—C7—C8	1.4 (5)	O2—Cd1—N3—C5	−178.35 (18)
C6—C7—C8—C9	0.6 (5)	S1^i^—Cd1—N3—C5	95.7 (2)
C7—C8—C9—N3	−1.8 (5)	C3—C4—N4—N5	−0.7 (4)
C7—C8—C9—N2	178.5 (3)	C3—C4—N4—C5	−176.5 (3)
N2—C10—C11—C12	−0.4 (4)	N3—C5—N4—N5	−2.6 (4)
C10—C11—C12—N1	0.3 (4)	C6—C5—N4—N5	178.0 (3)
C11—C12—N1—N2	−0.1 (4)	N3—C5—N4—C4	172.9 (3)
C11—C12—N1—Cd1	162.7 (3)	C6—C5—N4—C4	−6.6 (5)
N6—Cd1—N1—C12	90.2 (3)	C3—C2—N5—N4	−0.3 (4)
O3—Cd1—N1—C12	−3.6 (4)	C3—C2—N5—Cd1	175.3 (2)
N5—Cd1—N1—C12	−177.6 (3)	C4—N4—N5—C2	0.6 (3)
N3—Cd1—N1—C12	−169.9 (4)	C5—N4—N5—C2	176.9 (3)
O2—Cd1—N1—C12	9.4 (3)	C4—N4—N5—Cd1	−176.07 (19)
S1^i^—Cd1—N1—C12	−83.1 (3)	C5—N4—N5—Cd1	0.2 (3)
N6—Cd1—N1—N2	−108.5 (2)	N6—Cd1—N5—C2	−72.3 (3)
O3—Cd1—N1—N2	157.76 (18)	N1—Cd1—N5—C2	−166.3 (3)
N5—Cd1—N1—N2	−16.3 (3)	O3—Cd1—N5—C2	17.8 (3)
N3—Cd1—N1—N2	−8.6 (2)	N3—Cd1—N5—C2	−174.1 (3)
O2—Cd1—N1—N2	170.7 (2)	O2—Cd1—N5—C2	2.8 (4)
S1^i^—Cd1—N1—N2	78.2 (2)	S1^i^—Cd1—N5—C2	103.4 (3)
C12—N1—N2—C10	−0.1 (4)	N6—Cd1—N5—N4	102.9 (2)
Cd1—N1—N2—C10	−166.9 (2)	N1—Cd1—N5—N4	9.0 (3)
C12—N1—N2—C9	178.6 (3)	O3—Cd1—N5—N4	−166.94 (19)
Cd1—N1—N2—C9	11.9 (3)	N3—Cd1—N5—N4	1.21 (18)
C11—C10—N2—N1	0.4 (4)	O2—Cd1—N5—N4	178.12 (16)
C11—C10—N2—C9	−178.3 (3)	S1^i^—Cd1—N5—N4	−81.36 (19)
N3—C9—N2—N1	−7.0 (4)	O1—N7—O2—Cd1	−177.4 (3)
C8—C9—N2—N1	172.7 (3)	O3—N7—O2—Cd1	2.5 (3)
N3—C9—N2—C10	171.5 (3)	N6—Cd1—O2—N7	95.51 (19)
C8—C9—N2—C10	−8.8 (5)	N1—Cd1—O2—N7	−170.26 (18)
C8—C9—N3—C5	0.9 (4)	O3—Cd1—O2—N7	−1.52 (16)
N2—C9—N3—C5	−179.3 (2)	N5—Cd1—O2—N7	17.5 (2)
C8—C9—N3—Cd1	178.9 (2)	N3—Cd1—O2—N7	−168.76 (17)
N2—C9—N3—Cd1	−1.3 (3)	S1^i^—Cd1—O2—N7	−84.41 (17)
C6—C5—N3—C9	1.3 (4)	O1—N7—O3—Cd1	177.2 (3)
N4—C5—N3—C9	−178.2 (3)	O2—N7—O3—Cd1	−2.7 (3)
C6—C5—N3—Cd1	−176.8 (2)	N6—Cd1—O3—N7	−77.21 (19)
N4—C5—N3—Cd1	3.7 (3)	N1—Cd1—O3—N7	17.9 (2)
N6—Cd1—N3—C9	94.7 (2)	N5—Cd1—O3—N7	−166.34 (18)
N1—Cd1—N3—C9	5.3 (2)	N3—Cd1—O3—N7	168.29 (17)
O3—Cd1—N3—C9	−153.0 (2)	O2—Cd1—O3—N7	1.52 (16)
N5—Cd1—N3—C9	179.3 (2)	S1^i^—Cd1—O3—N7	97.59 (17)
O2—Cd1—N3—C9	3.7 (3)	N6—C1—S1—Cd1^ii^	179 (100)
S1^i^—Cd1—N3—C9	−82.3 (2)		

Symmetry codes: (i) −*x*+3/2, *y*−1/2, −*z*+1/2; (ii) −*x*+3/2, *y*+1/2, −*z*+1/2.

### Hydrogen-bond geometry (Å, °)

**Table:**

*D*—H···*A*	*D*—H	H···*A*	*D*···*A*	*D*—H···*A*
C3—H3···O1^iii^	0.93	2.50	3.412 (5)	167
C4—H4···O2^iv^	0.93	2.47	3.370 (4)	164
C7—H7···O3^v^	0.93	2.52	3.312 (5)	143
C10—H10···S1^v^	0.93	2.83	3.723 (4)	160

Symmetry codes: (iii) −*x*+1/2, *y*+1/2, −*z*+1/2; (iv) *x*−1/2, −*y*+3/2, *z*−1/2; (v) *x*+1/2, −*y*+3/2, *z*−1/2.
